# Primary biliary cirrhosis and autoimmunity: molecules and mimics

**DOI:** 10.1186/1479-5876-10-S2-A24

**Published:** 2012-10-17

**Authors:** M Eric Gershwin

**Affiliations:** 1 Division of Rheumatology, Allergy and Clinical Immunology, University of California at Davis School of Medicine, 451 Health Sciences Drive, Suite 6510, Davis, CA 95616, USA

## 

There have been significant advances both in humans and experimental models that relate to the etiopathogenesis of primary biliary cirrhosis. Many of these advances are based on the rigorous definition of the antimitochondrial response, the serologic signature of PBC. First, it is well established that AMA are directed against members of the 2-oxoacid dehydrogenase complexes (2-OADC), among which the major epitopes are within the lipoylated domains of the E2 subunit of the pyruvate dehydrogenase complex (PDC-E2). Second, autoreactive CD4+ and CD8+ T cells can be detected in PBC peripheral blood, regardless of the AMA status, and the infiltration of autoreactive T cells in the liver and periductular spaces is one of the most prominent immune features. Autoreactive T cells of both subtypes recognize PDC-E2 sequences overlapping with the AMA epitopes. An increase in cytotoxic T cell precursors in the blood in the early stages of the disease compared to the advanced ones and a 10-fold increase of specific liver CD8+ T cells compared to peripheral blood have been demonstrated. Third, additional data on the immunobiology components of PBC autoimmunity has been recently obtained in CD4+CD25 ^high^ natural regulatory T cells which appear to be numerically reduced in PBC. PBC bile duct cells manifest unique features during apoptosis while co-culture experiments do not support a direct role for these cells in determining their immune –mediated injury. Apoptotic cells are phagocytosed by BECs and consequently are an exogenous source of autoantigens in cholangiocytes, possibly through anti-CD16. As a result, the impact of putative changes in apoptosis and autophagy specific to BEC remains to be fully determined in PBC. Fifth, the innate immune compartment has been recently investigated in PBC with promising results. PBC monocytes manifest an increased response to pathogen associated stimuli, as indicated by higher levels of pro-inflammatory cytokines. Further, the hyper-IgM associated with PBC is secondary to an aberrant innate immune response, potentially induced by stimulation of toll like receptor 9 by bacterial CpG-B.

The female preponderance may hold an important key to PBC etiology. X-linked genes determine gender-related characteristics at different levels while also regulating the immune function, particulalry to maintain tolerance. Major X chromosome defects such as those leading to Turner’s syndrome or premature ovarian failure are commonly characterized by autoimmune comorbidities (particularly thyroid disease) and, less frequently, cholestasis. Our group first determined a significantly higher frequency of monosomy of the X chromosome in peripheral leukocytes (particularly those of the adaptive immune response, i.e. T and B cells) in women PBC compared to age-matched control women. Monosomy frequency correlated with age in all three groups, as expected but monosomic cells were not microchimeric cells. We further demonstrated that the X loss in PBC affected was not random but affected more frequently one parentally-inherited chromosome.

Several key animal models of autoimmune cholangitis have now been described. First, a genomic variant of the non obese diabetic (NOD) mouse (NOD.c3c4) has been observed to manifest autoimmune cholestasis with AMA and ANA positivities in 50%–60% and 80%–90%, respectively. Liver histology demonstrated portal lymphocyte infiltration with chronic non-suppurative cholangitis and PBC-like granulomas. Second, a dominant negative form of trasforming growth factor β (TGFβ) receptor II (dnTGFβRII) mouse develop serum AMA in 100% of mice. The TGFβ receptor II regulates lymphocyte activation and the appearance of PBC in this model suggests a specific condition of T cells with impaired TGFβ signaling in the presence or absence of B cells is involved. Third, the knockout of interleukin 2 receptor α leads to a murine phenotype with 100% serum AMA positivity, 80% serum ANA positivity, and portal lymphocyte infiltration and vanishing bile ducts. This model is of particular interest based on the report of autoimmune cholangitis in a pediatric case of IL2Rα deficiency. Fourth, Ae2a,b also develop autoimmune phenomenon and a PBC-like disease. Finally, immunization of mice with chemical xenobiotics has also been shown to lead to a PBC-like disease.

These data and observations will be put in the context of the key mechanisms, including the role of TLRs in modulating these responses.

